# Supplementary material for: Stress Effects on Temperature-Dependent In-Plane Raman Modes of Supported Monolayer Graphene Induced by Thermal Annealing
Source: Nanomaterials (Basel). 2021 Oct 17;11(10):2751. doi: 10.3390/nano11102751 (PMC8538804; doi:10.3390/nano11102751)
Supplement: Supplementary file 1 [file nanomaterials-11-02751-s001.zip › nanomaterials-1400119-supplementary.pdf]

Supporting Information

Stress Effects on Temperature-Dependent in-Plane Raman Modes of Supported Monolayer Graphene Induced by Thermal Annealing

Yuehua Wei [1], Zhenhua Wei [2], Xiaoming Zheng [3], Jinxin Liu [3], Yangbo Chen [3], Yue Su [3], Wei Luo [2], Gang Peng [2], Han Huang [4], Weiwei Cai [3], Chuyun Deng [2,*], Xueao Zhang [3,*] and Shiqiao Qin [1,*]

[1] College of Advanced Interdisciplinary Studies, National University of Defense Technology,
Changsha 410073, China; 15116271019@163.com

[2] College of Arts and Sciences, National University of Defense Technology, Changsha 410073, China; weizhsx@139.com (Z.W.); luoweihust@163.com (W.L.); 13507480737@126.com (G.P.)

[3] College of Physical Science and Technology, Xiamen University, Xiamen 361005, China;
zhengxiaoming2020@xmu.edu.cn (Xiaoming Zheng); jxliu@stu.xmu.edu.cn (J.L.); bobby-chen@stu.xmu.edu.cn (Y.C.); suyuelnu@163.com (Y.S.); wwcai@xmu.edu.cn (W.C.)

[4] Hunan Key Laboratory of Super-microstructure and Ultrafast Process, School of Physics and Electronics, Central South University, Changsha 410083, China; physhh@csu.edu.cn

* Correspondence: dengchuyun@nudt.edu.cn (C.D.); xazhang@xmu.edu.cn (Xueao Zhang); sqqin8@nudt.edu.cn (S.Q.)

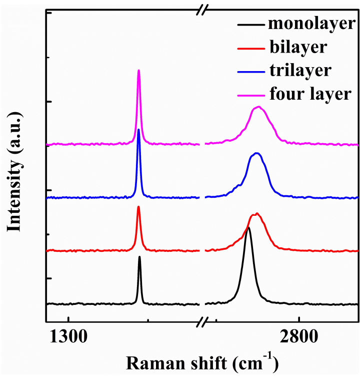

Figure S1. The Raman spectra of different layer graphene.

Table S1. the full width at half maximum (FWHM) of 2D band for different layer graphene.

Graphene layer	The FWHM of 2D band (cm^{-1})
monolayer	~30
bilayer	~54
trilayer	~59
four layer	~61

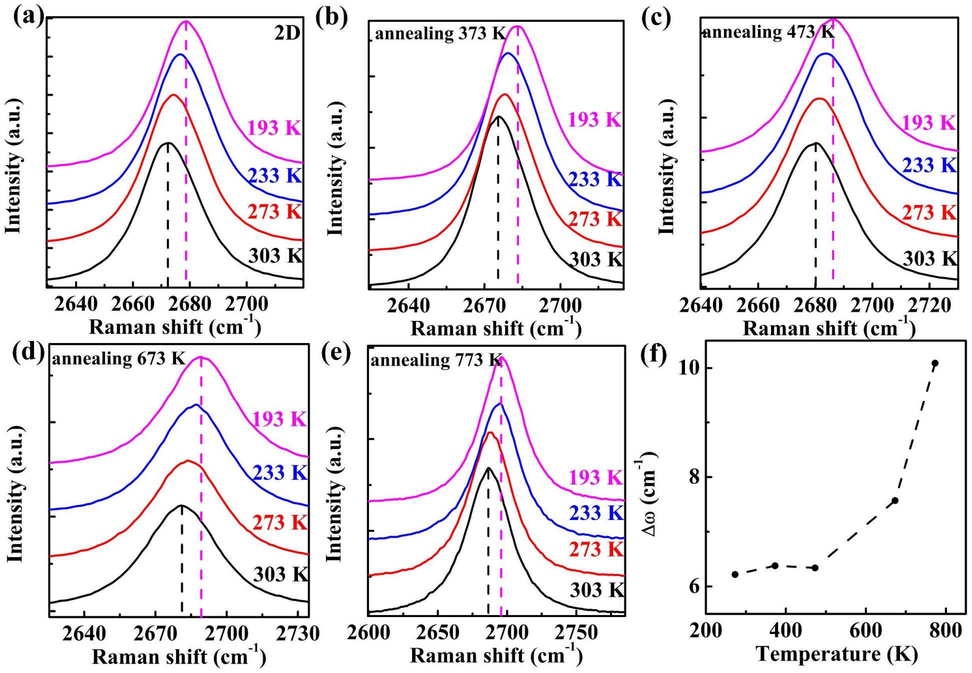

Figure S2. The temperature-dependent Raman spectra of 2D band for supported monolayer graphene flakes with various thermal annealing processes. (**a**) Pristine; (**b**) Annealing at 373 K; (**c**) Annealing at 473 K; (**d**) Annealing at 673 K; (**e**) Annealing at 773 K; (**f**) The evolution of Raman peak redshift for 2D band as a function of thermal annealing temperature.

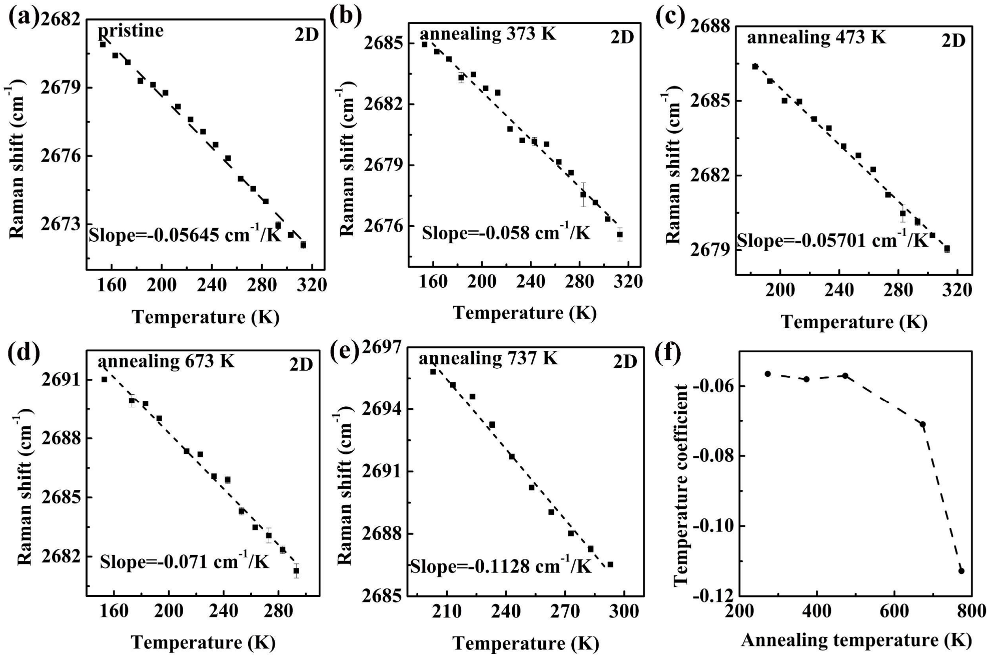

Figure S3. The temperature coefficients of 2D band for supported monolayer graphene flakes with various thermal annealing processes. (**a**) Pristine; (**b**) Annealing at 373 K; (**c**) Annealing at 473 K; (**d**) Annealing at 673 K; (**e**) Annealing at 773 K; (**f**) The extracted temperature coefficients of monolayer graphene under different annealing temperatures.

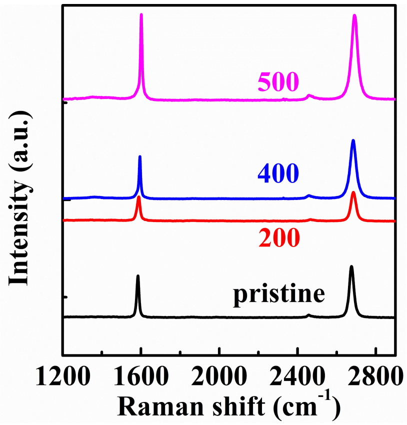

Figure S4. The Raman spectra of supported monolayer graphene with various thermal annealing process.

Table S2. The summary of Raman peak shift.

Thermal annealing temperature (K)	G band peak shift (cm^{-1})	2D band peak shift (cm^{-1})
373	0.45	5.1
473	6.13	8.66
673	9.11	8.72
773	15.09	15.06

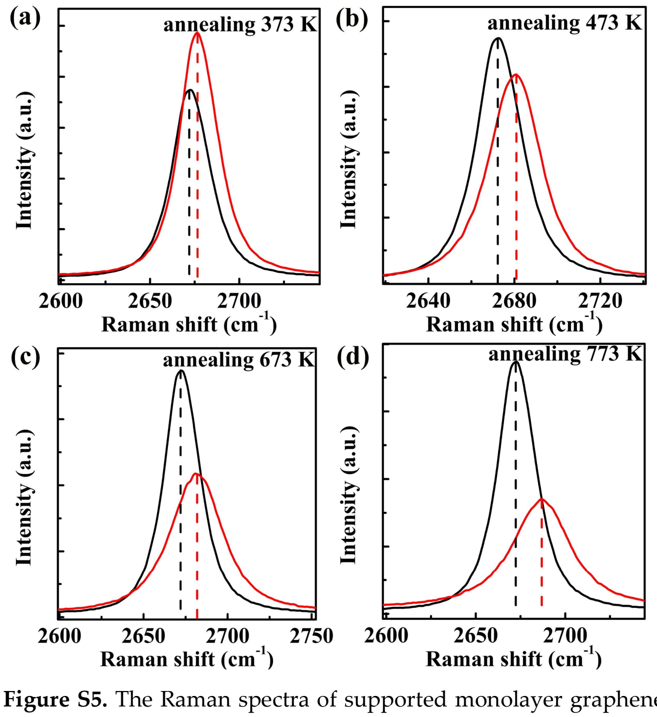

Figure S5. The Raman spectra of supported monolayer graphene after thermal annealing at different temperatures. (**a**–**d**) The Raman spectra annealing at 373 K, 473 K, 673 K, 773 K.

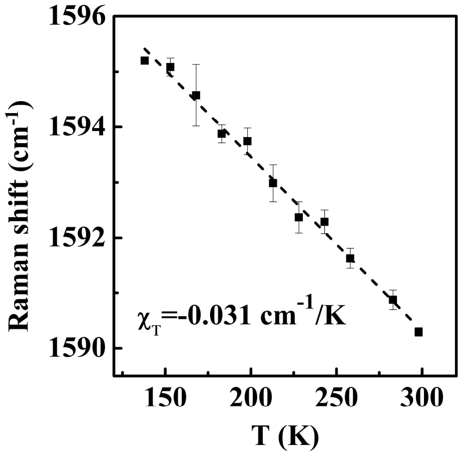

Figure S6. The temperature-dependent Raman spectra of monolayer graphene on BN.
